# Trends, prevalence and determinants of childhood chronic undernutrition in regional divisions of Bangladesh: Evidence from demographic health surveys, 2011 and 2014

**DOI:** 10.1371/journal.pone.0220062

**Published:** 2019-08-09

**Authors:** Unnati Rani Saha, Aparajita Chattapadhayay, Jan Hendrik Richardus

**Affiliations:** 1 Department of Public Health, Erasmus MC, University Medical Center Rotterdam, Rotterdam, The Netherlands; 2 International Institute for Population Sciences (IIPS), Mumbai, India; University of Zurich, SWITZERLAND

## Abstract

**Background:**

Undernutrition, an important indicator for monitoring progress of development goals, is a matter of concern in many developing countries, including Bangladesh. Despite regional differences in chronic undernutrition in Bangladesh, regional determinants among children under the age of five were not extensively explored.

**Data and methods:**

Using combined repeated cross-sectional nationwide Bangladesh Demographic and Health Surveys (BDHS 2011 and 2014) and employing bivariate and logistic regression analyses, we estimated prevalence, changes and variations in regional determinants of stunting among children aged 6–59 months over two time periods 2011 and 2014.

**Results:**

Our benchmark results suggested that the children from Rajshahi, Khulna, Rangpur, Chittagong and Dhaka tend to be significantly less stunted by 51% (p = 0.000; CI = [0.38, 0.63]), 44% (p = 0.000; CI = [0.44, 0.71]), 26% (p = 0.012; CI = [0.58, 0.93]), 23% (p = 0.012; CI = [0.62, 0.95]) and 22% (p = 0.033; [0.63, 0.97]) respectively, against Sylhet in 2011. With the exception of Dhaka, no region showed significant differences in the odds of stunting over two time periods 2011 and 2014, i.e. only Dhaka revealed significant difference by 30% reductions in the odds of stunting in 2014. Also, rural children were less likely to be stunted (by 19%) of the urban counterparts. Regional covariates of stunting differ. However, children’s age, household wealth, mother’s height, and parental education were important determinants of stunting in Bangladesh.

**Conclusion:**

Dhaka made an impressive improvement in child nutrition, thus contributed largely to the reduction of stunting levels in Bangladesh for 2014 over 2011. Sylhet and Barisal require strong push to improve nutritional status of children. Further decline is possible through region-specific multipronged interventions that can address area-specific covariates to break the cycle of undernutrition like strengthening economic and educational status, emphasizing the role of father to augment their knowledge in varying aspects like family planning, reduction of fertility and by improving mother’s health.

## Introduction

Globally, nutritional status measured in height-for-age or stunting is considered as one of the best predictors of well-being for young children [1]. It is an important indicator for monitoring progress towards the second Sustainable Development Goal that aims to terminate all forms of malnutrition by 2030 [2–3]. Undernutrition continues to be a burden in many developing countries and Bangladesh is not an exception. Bangladesh is one of the signatories of the Sustainable Development Goals (SDG) committed to achieve the SDG target of reducing the proportion of stunting from prevailing levels (2014 est.) to 25% by 2030 [4].

The World Health Organization has identified undernutrition as the underlying cause for almost half of the child deaths worldwide [5–6]. In developing countries, about 13 million children under the age of five die annually and many of them are linked to malnutrition [7]. There is strong evidence that children who are suffering from linear growth failure are more likely to be affected by infectious diseases, such as, malaria, diarrhoea and pneumonia [8]. Stunting affects children, not only by causing large number of deaths among them and making them more susceptible to disease, but it also negatively influences children’s behaviour. Poor nutrition during childhood causes irreversible damage to cognitive development and future health. Strong associations are observed on analysing child malnutrition along with fewer years of schooling and reduced economic productivity [9–10]. Grantham *et al*., [11] in 2007 estimated that 2.91 years of performance deficit that resulted from stunting caused 19.8% loss in adult income. Thus, damage caused in early life can affect future generations and the cycle of undernutrition recapitulates. Improving nutrition can have a significant impact on survival as well as physical and cognitive development and productivity. Therefore, from a policy perspective, it is imperative to understand stunting prevalence and its determinants at various levels, i.e. individual, household, residential and regional.

Levels, trends and determinants of stunting in children under-five years are well established [12, 13]. For instance, certain vital predictors of stunting in Bangladesh are parental education, maternal nutrition, preceding short birth interval, socioeconomic status, size of the baby at birth, birth order and place of residence. Studies have consistently revealed regional differences in the prevalence of stunting (height-for-age) is more than two standard deviations below the WHO Child Growth Standards median developed by the World Health Organization. However, what is not explored yet is the detailed regional prevalence of stunting vis-a`-vis socio-economic groups and other possible covariates.

The physical features of Bangladesh is varied and characterized by two distinctive features: a broad deltaic plain subject to frequent flooding, and small hilly areas i.e. Chittagong Hills in the southeast, the Low Hills of Sylhet in the northeast, and highlands in the north and northwest. The high population density mainly in the plain lands has led to land saturation for agricultural expansion. While, climate change and infrastructural bottlenecks of basic amenities do have intense varying impact on production, availability and accessibility of food in different divisions of Bangladesh. In such case of wide regional it is meaningful to understand the undernutritional aspects of seven disparate divisions when determinants of stunting are largely context specific.

We investigated the prevalence of stunting and their unadjusted risk factors among children under the age of five in different regions of Bangladesh. We also built models to examine how covariates play a role in determining urban-rural and regional variances in the prevalence of stunting over these two periods (2011 and 2014). Findings may help influence emerging policies related to child nutrition and development in Bangladesh.

## Data and methods

Two terms i.e. undernutrition and stunting are interchangeably used in the research paper. Bangladesh Demographic Health Survey (BDHS) 2011 and 2014 are nationally representative surveys, which gathered information on a wide range of socio-demographic and health indicators. Both the rounds of BDHS (2011 and 2014) were cross-sectional surveys with two-stage stratified sampling design. Census enumeration areas were selected as primary sampling unit at the first stage of the sampling frame. At the second stage, households were randomly selected from the primary sampling unit ‘çluster’ of the sampling frame. For details about the sampling technique we refer [14–15].

**Fig 1:**
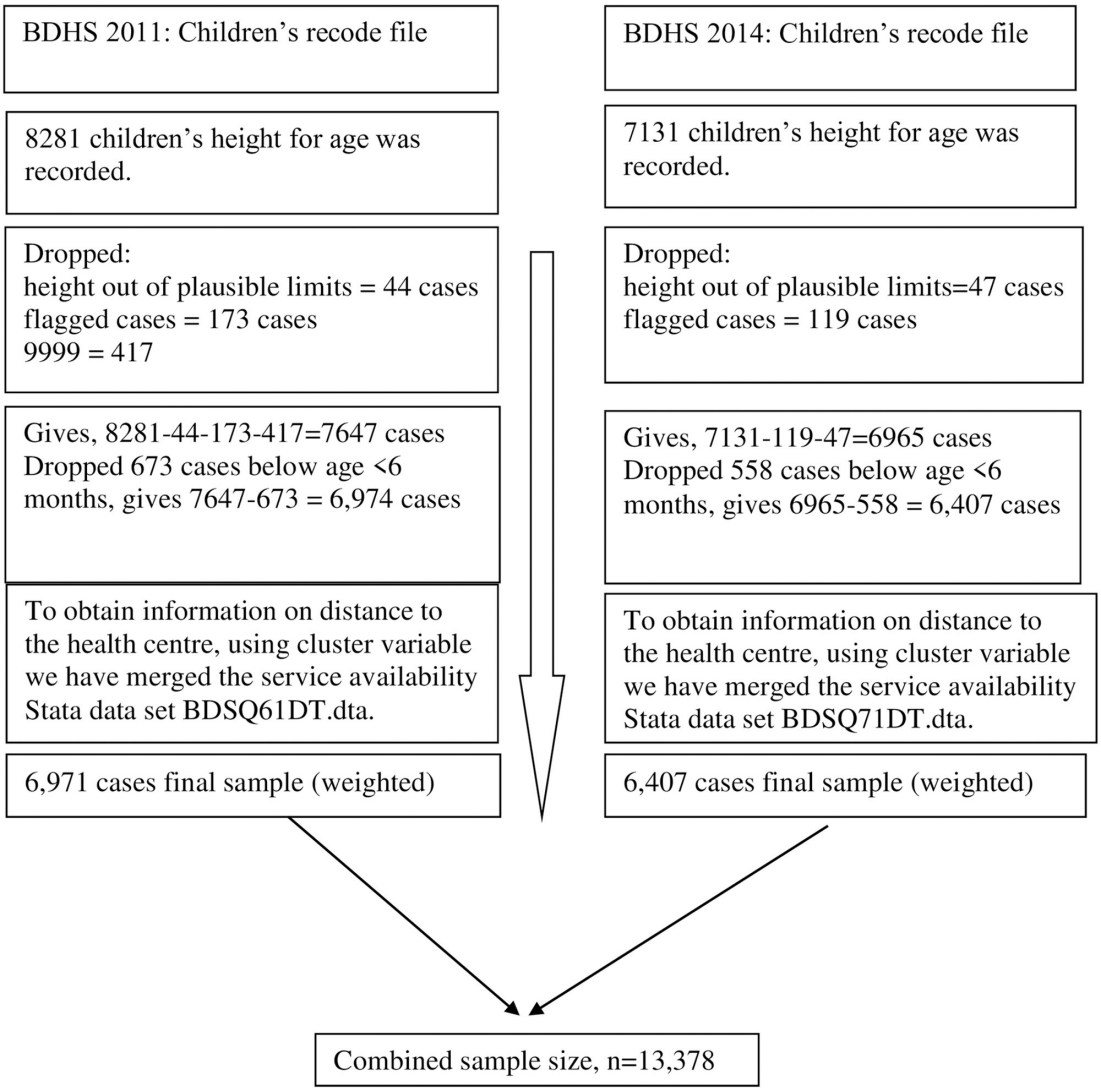
Data File and Sample flow.

Before attempting our main analysis, we examined the trend of stunting for the period 1996 to 2014 based on the prevalence measured by BDHS in its 6 consecutive data sets (preliminary report BDHS 1996–97 to BDHS 2014). Rangpur division, formed in 2010 as Bangladesh's 7th division, was basically a part of Rajshahi division. Two consecutive BDHS 2011 and BDHS 2014 data sets were then used mainly to comprehend the extent of change in prevalence of stunting over time in heterogeneous regions and to understand the variation of regional determinants. The measurement of linear physical growth, i.e. height-for-age (stunting) score as an indicator of chronic nutritional status, was available in the data set. The dependent variable for this study was moderate stunting (HAZ<-2 SD), i.e. child who was more than two standard deviations below the median (-2 SD) of the WHO reference population in terms of height-for-age (HAZ) referred to 1, otherwise 0. The study sample included 6,974 and 6,407 children aged 6–59 months from 6,190 and 5,819 mothers for BDHS 2011 and BDHS 2014, respectively. For logistic regression analyses to investigate the covariate adjusted regional stunting over two periods, we combined both data sets (N = 13,378). The data files and sample flows were given in Fig 1.

The exclusion criteria based on the World Health Organization (WHO) 2006 standards the flagged codes were applied to measure implausible values when the components are present in the construction variables but implausible either individually or in combination. When a case is flagged, it is impossible to identify with the available information whether an error resulted in the field as the measurements were recorded or in the office as they were entered in the computer file. The cases were assigned implausible for which HAZ score was less than -600 or greater than +600, that is, more than six standard deviations away from the median normative height.

Taking into account different literature on stunting, variables considered as covariates in this study were sets of child, parent, household and community level variables. Variables that capture child’s characteristics were gender and age of a child, birth order, religious affiliation of child and administrative division or region of residence. Variables considered for mother’s characteristics were schooling years, maternal nutritional indices and maternal age at child birth. Household wealth quintiles represent household economic condition was based on the available wealth score in the original BDHS datasets. Father’s schooling was entered in the model as a proxy for social status of household. The community level variable was the distance to the nearest health centre. This variable was computed using variables on distance (converted mile in kilometre) to the government authorities: hospital, *Thana* (local police station) Health Complex (THC), Family Welfare Centre (FWC) and Maternal and Child Welfare Centre (MCWC). We replaced distance to hospital variable by the distance to THC if distance to THC is less than the distance to hospital; otherwise it remains the value of hospital. This procedure was repeated for the distance of FWC and MCWC as well.

Chi square test was used to summarize the significant levels of unadjusted covariate’s effect on level of stunting in different regions. Multiple logistic regression analyses were applied in 3 models to examine regional differences and covariate effects on the stunting outcome between two time periods as captured by introducing time dummy and interaction terms (time dummy with region dummies and time dummy with type of residence). The time dummy takes code 1 if variables were measured in 2014, otherwise 0 for variables in 2011. Model 1 takes into account the effect of regions along with regional interactions. Model 2 included child and maternal level characteristics in addition to the variables in model 1. Model 3 revealed the odds ratios of variables where household wealth, father’s education and distance to nearest health facility were also included apart from the variables already mentioned in model 1 and 2. Further, we had also estimated another model, i.e. model 4 that revealed the odds ratios of all variables in addition to interaction terms with time dummy and all child, maternal and HH level factors. However, no interaction terms were found significant except regional interactions. Therefore, we have not reported the results of model 4 in our results section under the sub-heading: Determinants of stunting over time: 2011 and 2014 (results are available upon request). Analyses were adjusted for primary sampling unit cluster with clustering specific effects and sampling weight.

### Ethical issue

Both surveys BDHS 2011 and 2014 were conducted under the authority of the National Institute of Population Research and Training (NIPORT) of the Ministry of Health and Family Welfare. Ethical approval for the BDHS 2011 and 2014 were taken by the NIPORT from the National Research Ethics Committee of the Bangladesh Medical Research Council (Dhaka, Bangladesh). ICF Macro Institutional Review Board (USA), which complied with all requirements of 45 CFR 46 “Protection of Human Subjects” had reviewed and approved both surveys. Informed consent was obtained from each respondent before enrolling in the survey. Since both rounds of survey included very young children, mothers of these samples were asked to provide verbal consent on behalf of their children.

## Results

### Trends in stunting by region

**Fig 2:**
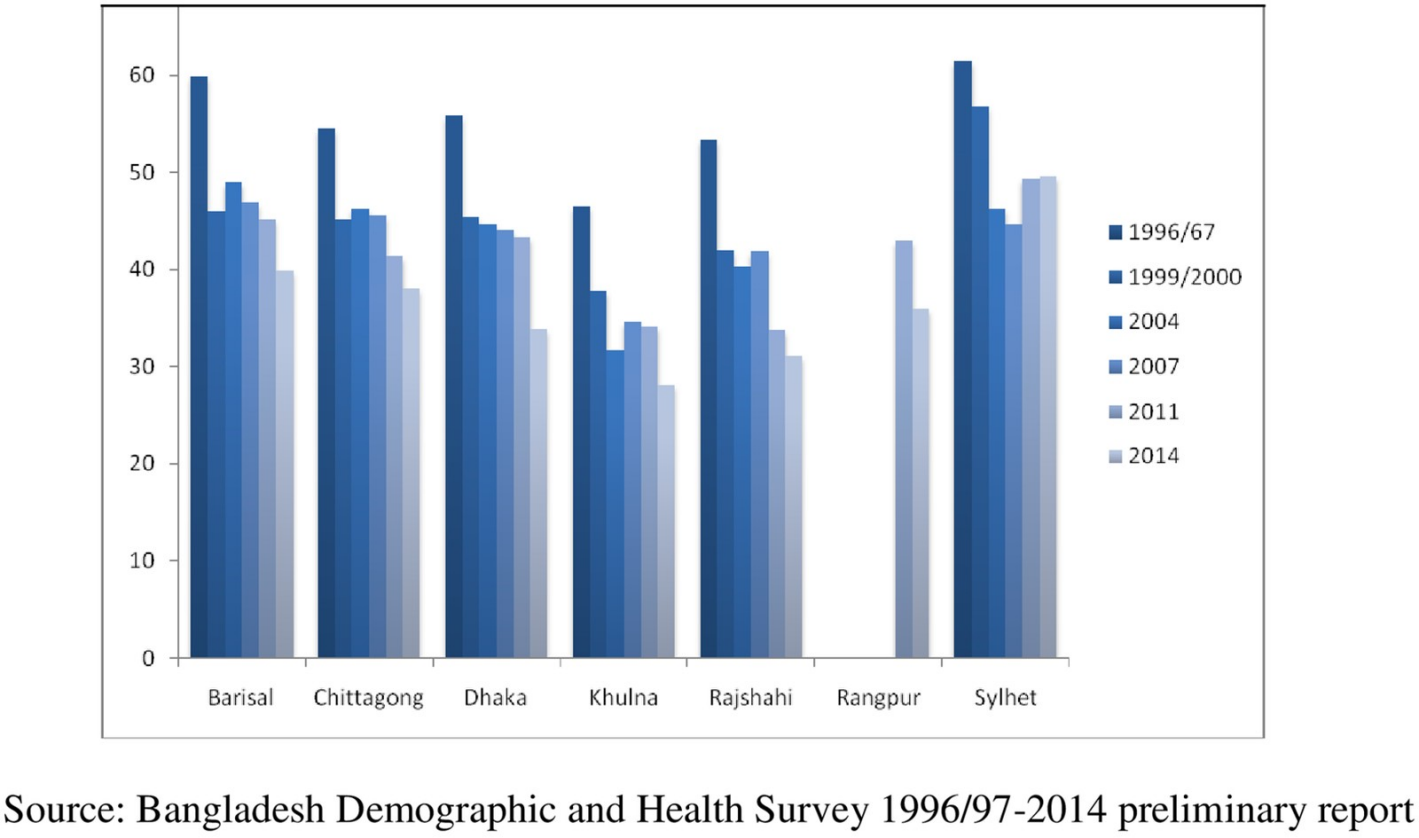
Trend of Stunting (0–59 months old children) in Bangladesh by Regional Divisions, 1996/97-2014.

Over the period, Bangladesh has witnessed considerable reductions in childhood stunting from 54.6% in 1996–97 to 36.1% in 2014 (NIPORT et al 1996/97) [14]; NIPORT et al 2014) [15]. Fig 2 reveals that this reduction varied across different regional divisions. Substantial reduction of childhood stunting was observed in almost all divisions over the past one and half decade, except Sylhet, where stunting was increased since 2007. Stunting in Khulna division was the lowest in 2014, followed by Rajshahi and Dhaka. In Khulna and Rajshahi, stunting was below the national average 36% [15]. Further, a significant reduction of about 10% was observed in Dhaka division during the period 2011 to 2014, which contributed to a marginal reduction of 5% (41% in 2011 to 36% in 2014) in the overall stunting level in Bangladesh. The level of stunting in Sylhet division was found consistently high from 61% in 1996–97 to 49.6% in 2014, which was significantly above the national average of 36% in 2014. Barisal and Chittagong, at stunting level of 42% and 41% respectively, also drew attention for policy interventions.

### Prevalence of stunting by region: 2011 and 2014

Tables 1 and 2 illustrated the unadjusted covariate effects on the prevalence of stunting and their significant levels for 2011 and 2014 respectively. Although prevalence of stunting for the age group 6–11 months was considerably lower as compared to other age groups (except Rajshahi) in 2011, no such marked variation was observed among the divisions in 2014. Children in the age group 48–59 months showed relatively improved growth in Khulna followed by Rajshahi. While conditions were the worst in Sylhet division with a stunting rate of 52.3% in 2011 and 52.0% in 2014. Considering religious affiliation, marginal differences were noticed in 2011, where Muslims were more stunted as compared to non-Muslim counterparts (p< = 0.05). However, significant religious differences were not observed in 2014. The urban-rural place of residence was considerably associated with stunting differentials in both the time periods, particularly in Dhaka, Sylhet, Barisal (p< = 0.001) in 2011 and in Chittagong (p< = 0.01), Rangpur (p< = 0.05) in 2014.

**Table 1:** Prevalence of stunting at 6–59 months old children by sample characteristics and birth regions (divisions) of Bangladesh, 2011.

Covariates	Dhaka, n = 1,161	Khulna, n = 794	Rajshahi, n = 834	Chittagong, n = 1,372	Rangpur, n = 912	Barisal, n = 778	Sylhet, n = 1,121	Total N = 6971
**Stunting (HAZ<-2 SD) age 6–59 months old children**	45.58	35.48	35.37	44.36	43.95	46.37	52.32	44.48
**Gender of child**								
Male	43.00	36.89	36.07	43.41	46.65	44.65	49.75	42.70
Female	48.31	34.14	34.63	45.29	41.01	48.18	54.97	44.28
**Age of child (months)**								
6–11	20.22***	14.88***	29.27	17.09***	30.39***	27.82***	31.58***	22.56***
12–23	54.28	46.94	39.55	45.00	54.71	52.93	54.94	49.37
24–35	46.43	32.32	39.17	56.19	47.66	46.16	57.92	47.42
36–47	50.11	42.23	35.68	49.18	43.33	54.12	53.77	47.14
48–59	44.10	30.23	30.79	44.08	40.19	41.43	53.52	41.42
**Birth order**								
1	43.89***	32.86	31.76	39.44***	41.85	41.97***	46.14	39.97***
2	42.49	36.50	33.98	43.84	48.61	41.19	50.48	42.06
3–4	44.32	36.92	39.25	46.32	41.60	52.27	55.75	44.09
5+	64.01	47.29	45.78	53.87	42.27	64.31	57.54	56.39
**Religion**								
Non-Muslim	41.45	25.73	19.04**	38.75	45.58	46.45	39.16*	37.86*
Muslim	45.75	36.69	36.44	44.85	43.58	46.36	54.77	44.00
**Type of residence**								
Urban	38.00**	32.37	29.81	45.22	41.42	33.03***	33.58***	38.2***
Rural	49.35	36.27	36.41	44.12	44.28	48.66	54.87	44.98
**Schooling years of Mother**								
No schooling year	53.37***	38.87	42.84***	55.33***	48.02	56.42***	66.49***	52.78***
Primary levels	52.66	37.59	38.79	54.80	45.91	54.69	55.03	49.62
Secondary+	36.88	34.08	29.55	34.99	40.84	38.37	36.91	35.71
**Mother’s BMI**								
>18.5 kg/m^2^	41.58***	32.75**	31.10***	39.86***	42.74	42.84*	46.26***	39.48***
< = 18.5 kg/m^2^	54.96	44.17	44.85	55.46	46.29	53.81	61.13	52.51
**Mother’s height**								
>145 cm	42.03***	33.21***	32.13***	41.28***	41.72**	43.10***	50.01***	40.41***
< = 145 cm	69.71	60.27	57.15	64.99	56.63	70.09	68.22	64.64
**Mother’s age at birth**								
> = 20 years	44.94	33.40	34.20	43.57	42.76	45.31	51.84	42.70***
<20 years	47.00	39.35	37.82	46.23	45.78	48.55	54.13	45.16
**Schooling years of Father**								
No schooling year	49.55***	44.39***	38.17***	50.16***	49.66***	53.27***	52.32***	48.38***
Primary levels	42.39	28.97	32.55	35.16	43.04	38.62	38.56	37.45
Secondary+	27.30	24.79	15.09	29.16	23.25	25.14	33.87	25.85
**Household wealth quintals**								
1^st^ quintile	56.99***	46.70***	44.76***	61.16***	52.43	66.77***	69.07***	56.42***
2^nd^ quintile	47.28	36.44	43.81	48.45	47.65	49.45	62.63	47.46
3^rd^ quintile	49.65	35.47	35.85	41.91	50.18	44.27	49.15	44.67
4thquintile	44.69	35.94	25.07	35.74	38.57	38.57	42.58	38.26
5^th^ quintal	26.89	18.51	18.69	29.46	25.01	22.95	26.77	25.44

* p< = 0.05;

** p< = 0.01

*** p< = 0.001

**Table 2:** Prevalence of stunting at 6–59 months old children by sample characteristics and birth regions (divisions) of Bangladesh, 2014.

Covariates	Dhaka n = 1,120	Khulna n = 711	Rajshahi n = 812	Chittagong, n = 1,215	Rangpur n = 791	Barisal n = 737	Sylhet n = 1,021	Total N = 6407
**Stunting (HAZ<-2 SD) age 6–59 months old children**	35.35	29.69	32.60	40.85	39.45	42.05	52.00	38.20
**Gender of child**								
Male	32.5	31.7	33.1	44.6**	42.5	42.5	54.0	38.7
Female	38.5	27.6	32.1	37.0	36.2	41.5	49.9	37.7
**Age of child (months)**								
6–11	19.6	18.6	20.7	18.0	24.9	17.0	18.6	19.5
12–23	36.8	39.9	27.4	41.8	39.1	37.1	50.3	38.4
24–35	36.5	27.7	40.4	39.7	48.0	53.3	58.4	41.3
36–47	42.6	29.8	36.8	51.0	39.9	49.2	67.2	45.3
48–59	34.8	26.3	33.8	45.0	37.8	43.1	50.5	38.7
**Birth order**								
1	27.2***	27.5	31.1	35.5**	38.3	36.6	50.1	33.0***
2	38.0	30.2	30.6	39.0	37.4	48.5	51.9	38.0
3–4	39.1	31.8	39.4	45.4	43.6	39.7	51.9	42.0
5+	56.6	41.4	25.9	54.5	48.4	53.5	53.6	52.8
**Religion**								
Non-Muslim	27.3	28.6	35.9	41.3	35.1	49.6	42.7	37.0
Muslim	35.7	29.8	32.4	40.8	40.0	41.1	53.0	38.3
**Type of residence**								
Urban	31.4	24.6	29.0	33.0**	30.7*	40.2	51.1	32.6***
Rural	37.3	31.4	33.4	43.8	40.8	42.6	52.2	40.1
**Schooling years of Mother**								
No education	47.0***	54.0***	35.3***	54.5***	36.4***	63.9***	57.1***	48.8***
Primary level	43.0	34.0	39.8	52.0	53.5	44.4	59.5	47.0
Secondary+	27.0	25.0	28.5	33.4	33.9	36.1	40.4	30.6
**Mother’s BMI**								
>18.5 kg/m^2^	32.3*	28.1	29.9**	39.2	37.9*	38.3**	49.6	35.7***
< = 18.5 kg/m^2^	45.8	36.3	39.5	47.1	44.9	51.9	57.3	46.4
**Mother’s height**								
>145 cm	32.9***	27.0***	27.9***	40.0***	35.6***	40.8	49.1***	35.2***
< = 145 cm	51.3	56.6	63.0	68.9	64.9	51.4	65.0	59.0
**Mother’s age at birth**								
> = 20 years	35.8	29.2	29.8*	42.1	35.9*	41.8	49.2**	37.9
<20 years	34.4	30.8	37.9	37.8	45.9	42.6	61.5	38.9
**Schooling years of Father**								
No education	46.8***	39.2**	42.9***	59.5***	43.0***	53.7**	59.1**	49.7***
Primary level	39.8	30.0	32.8	49.7	48.7	45.4	52.4	43.1
Secondary+	25.1	26.0	24.8	28.2	31.8	34.7	39.7	28.0
**Household wealth quintiles**								
First quintile	46.4***	42.2**	39.8***	58.1***	52.6***	55.6***	74.8***	51.7***
2^nd^ quintile	41.1	29.8	42.3	47.5	45.2	43.7	47.9	42.9
3rd quintile	37.9	28.0	32.2	39.0	42.4	44.0	56.4	39.2
4th quintile	26.7	27.7	24.1	30.8	37.4	36.0	42.4	30.7
5th quintile	16.5	18.1	17.7	23.3	17.3	28.1	27.6	20.1

* p< = 0.05;

** p< = 0.01

*** p< = 0.001

Percentage distribution of stunting varied significantly (p<0.001) by mother’s and father’s education, mother’s height and BMI kg/m^2^ in both 2011 and 2014. It was observed to be common in almost all divisions and in both the survey periods. Mother’s level of education helped in the reduction of stunting remarkably. For instance, when 49% children of illiterate mothers were stunted, the same is applicable for only 31% children whose mothers have secondary and above grade education (Table 2). Similarly, in 2014, about 35 percent children whose mothers were 145 cm or above in height were stunted, while similar condition was experienced by 59% children whose mothers were less than 145 cm. Another distinct factor that depicts broad variations in stunting was household wealth. When 20% and 25% children of the richest household were stunted in 2014 and 2011 respectively, higher levels of stunting were witnessed among children in the poorest household during that period (56% and 52%). Higher number of children under the age of five in household also leads to more stunting, irrespective of time.

### Determinants of stunting over time: 2011 and 2014

To distinguish the covariates adjusted regional and urban-rural differences between two time periods, we entered a time dummy, and interaction terms involving regional and urban-rural dummies in the multiple regression models. The time dummy measured odds of 2014, with reference to 2011. The odds ratios against interaction terms explained the changes in the odds of stunting in 2014 compared to the baseline odds of all regional and rural dummies in 2011. Our benchmark model was model 3 (see Table 3).

**Table 3:** Estimations from logistic regression results on all sample children from DHS 2011 and 2014, n = 13,378.

Covariates	Model 1	Model 2	Model 3
	Odds ratios (CIs)	Odds ratios (CIs)	Odds ratios (CIs)
**Birth regions**			
Barisal	0.79 [0.61, 1.04]	0.89[0.70, 1.148]	0.82[0.64, 1.05]
Chittagong	0.74*[0.58, 0.96]	0.84[0.67, 1.04]	0.77*[0.62, 0.95]
Khulna	0.51***[0.40, 0.66]	0.64***[0.50, 0.81]	0.56***[0.44, 0.71]
Rajshahi	0.50***[0.38, 0.66]	0.53***[0.41, 0.69]	0.49***[0.38, 0.63]
Rangpur	0.71**[0.56, 0.90]	0.76* [0.60, 0.97]	0.74**[0.58, 0.93]
Dhaka	0.81 [0.64, 1.04]	0.85[0.68, 1.06]	0.78*[0.63, 0.98]
**Type of residence**			
Rural	1.36***[1.15, 1.61]	1.17*[1.00, 1.37]	0.81*[0.69, 0.96]
**Survey year**			
DHS2014	1.01[0.71, 1.43]	0.98[0.71, 1.35]	1.04[0.75, 1.45]
**Interaction terms**			
DHS2014 Barisal	0.86[0.58, 1.28]	0.91[0.62, 1.31]	0.98[0.66, 1.43]
DHS2014 Chittagong	0.88[0.62, 1.26]	0.99[0.72, 1.36]	1.03[0.75, 1.41]
DHS2014Khulna	0.78[0.54, 1.14]	0.82[0.57, 1.16]	0.86[0.60,1.23]
DHS2014 Rajshahi	0.90[0.61,1.31]	0.98[0.69, 1.40]	0.98[0.69, 1.38]
DHS2014Rangpur	0.84[.57, 1.23]	0.95[0.64,1.40]	0.98[0.66, 1.45]
DHS2014 Dhaka	0.65* [0.45, 0.94]	0.71*[0.50, 0.99]	0.70*[0.50, 0.99]
DHS2014 Rural	0.99 [0.76, 1.27]	1.01 [0.79, 1.28]	1.01 [0.79, 1.29]
**Age of child (months)**			
12–23	-	3.24*** [2.70, 3.88]	3.30*** [2.74, 3.98]
24–35	-	3.23*** [2.71, 3.84]	3.30*** [2.75, 3.95]
36–47	-	3.53*** [2.94, 4.23]	3.63*** [3.02, 4.37]
48–59	-	2.59*** [2.15, 3.12]	2.63*** [2.17, 3.18]
**Gender of child**			
Male	-	1.00[0.92, 1.10]	1.00 [0.92, 1.10]
**Religion**			
Non-Muslim	-	0.89 [0.76, 1.03]	0.87 [0.74, 1.02]
**Birth order** ^a^		1.10***[1.06, 1.15]	1.07*** [1.03, 1.11]
**Mother’s age at birth**			
<20 years	-	1.33*** [1.18, 1.49]	1.19** [1.06, 1.33]
**Mother’s BMI**			
< = 18.5 kg/m^2^	-	1.48*** [1.30, 1.69]	1.33*** [1.16, 1.52]
**Mother’s height**			
< = 145 cm	-	2.61*** [2.27, 2.99]	2.45*** [2.14, 2.81]
**Schooling years of Mother**			
Primary levels	-	0.98 [0.83, 1.16]	1.07 [0.91, 1.26]
Secondary+	-	0.62*** [0.53, 0.72]	0.94 [0.79, 1.11]
**Schooling years of Father**			
Primary levels	-	-	0.89* [0.80, 1.00]
Secondary+	-	-	0.66*** [0.56, 0.76]
**Household wealth quintals**			
2^nd^ quintile	-	-	0.79** [0.68, 0.92]
3^rd^ quintile	-	-	0.74*** [0.64, 0.86]
4^th^ quintile	-	-	0.58*** [0.49, 0.68]
5^th^ quintile	-	-	0.37*** [0.29, 0.45]
**Distance to nearest health clinics** ^a^ **(kilo.)**	-	-	1.01 [1.00, 1.02]
**Log likelihoods**	**-9069.2108**	**-8505.3248**	**-8353.072**

* p< = 0.05;

** p< = 0.01

*** p< = 0.001; 95% confidence intervals (CIs) are within bracket.

Reference category: Sylhet division, urban residence, 6–11 months old children, female child, religious affiliation of child is Muslim, survey year 2011, 0 year of schooling, mother’s BMI >18.5 kg/m^2^, mother’s height>145 cm, HH wealth quintile 1^st^

^a^ = birth order and distance to the nearest health clinics is entered as continuous variable

model 1: regional dummies and regional interaction terms

model 2: child and maternal factors in addition to model 1

model 3: benchmark model which includes socioeconomic indicators, distance to the nearest health clinics in addition to model 1 & model 2

In all models, Sylhet was the reference category. Thus, in presence of all regional dummies including regional interaction terms in the models, the time dummy indeed captured the difference in the odds of stunting in Sylhet division between two years i.e. 2011 and 2014. No significant difference in the odds of stunting for children in Sylhet was estimated for 2014 compared to their baseline odds in 2011 (odds ratio = 1.0). This helps to interpret regional interaction effects straightforward without adjusting differences in the baseline odds.

Model 1 showed that in 2011 (baseline), children from Rajshahi, Khulna, Rangpur and Chittagong were significantly less likely to be stunted by 50% (p = 0.000; CI = [0.38, 0.66]), 49% (p = 0.000; CI = [0.40, 0.65]), 29% (p = 0.005; CI = [0.56, 0.90]), and 26% (p = 0.024; CI = [0.58, 0.96]) than those living in Sylhet. Interaction effects reveal the degree of effect of region between 2011 and 2014. Dhaka revealed significant difference by 35% (p = 0.023; [CI = 0.45, 0.94]) reductions in the odds of stunting in 2014. Also, in 2011, the likelihood of being stunted was significantly higher by 36% (p = 0.000; CI = [1.15, 1.61]) among rural children. Although model 1 and model 2 explained higher likelihood of stunting among rural children, such likelihood reversed when we adjusted model 3 (the benchmark model) for socioeconomic indicators in households and distance to the nearest health clinics. Rural children were significantly less stunted by about 19% (p = 0.017, CI = [0.69–0.96]). However, no significant changes in urban-rural differences in the odds of stunting were observed between 2011 and 2014.

Model 3 also suggested that the children from Rajshahi, Khulna, Rangpur, Chittagong and Dhaka tend to be significantly less stunted by 51% (p = 0.000; CI = [0.38, 0.63]), 44% (p = 0.000; CI = [0.44, 0.71]), 26% (p = 0.012; CI = [0.58, 0.93]), 23% (p = 0.012; CI = [0.62, 0.95]) and 22% (p = 0.033; [0.63, 0.97]) respectively, against Sylhet in 2011. Except for Dhaka, no region showed significant differences in the odds of stunting over two time periods 2011 and 2014, i.e. only Dhaka revealed significant difference by 30% reductions in the odds of stunting in 2014.

Among risk factors of stunting in model 3, maternal and child level factors and household (HH) asset quintiles, were found to have profound role in determining the overall prevalence of stunting among under-fives. For example, if the mother was taller than 145 cm and the body mass index was higher than 18.5 kg/m^2^, then it was probable that their children will be significantly less likely to be stunted. Similarly, richest quintile explained about 63% reductions in the odds of stunting. Approximately, there was a 19% higher chance for children to be shorter in future when born to a young mother aged less than 20 years. Similarly, higher the father’s schooling, it was significantly less likely for the child to be stunted. The child’s age and birth order variables were linearly associated with the child’s growth.

### Determinants of stunting in seven divisions over time

Table 4 explained variations in regional determinants of stunting among children aged 6–59 months. Apart from Dhaka, no significant reduction in stunting between two survey periods, i.e. 2011 and 2014 was observed in any of the divisions in Bangladesh. This finding is in line with our finding in Table 3. An increase in child’s age was clearly linearly associated with stunting, especially in Sylhet and Chittagong. Non-Muslim households had higher chances of being less stunted, especially in Sylhet and with reverse association in Barisal. Mother’s health, mainly better height, act as one of the key determinants in reducing stunting in all divisions of Bangladesh; the odds ratios, p-values, and CIs for height below 145 cm in seven regions were as follows: Dhaka: 2.32; p = 0.000; [1.71. 3.14], Khulna: 3.46; p = 0.0001; [2.34, 5.11], Rajshahi: 3.26; p = 0.000; [2.42, 4.39], Chittagong: 2.78; p = 0.000; [2.15, 3.59], Barisal: 2.13; p = 0.000; [1.43, 3.17], Rangpur: 2.18; p = 0.000; [1.58. 3.01], and Sylhet: 2.09; p = 0.000; [1.52, 2.89], respectively. Father’s education, particularly if it was above secondary, played an active role in reducing stunting in Rajshahi, Dhaka, Chittagong while mother’s education had such strong association in Sylhet. An improvement in household economic status immensely reduced stunting in all regions. However, in Dhaka, Chittagong and Rajshahi, only the households that were among the middle and above middle wealth quintile marked a noteworthy improvement in stunting.

**Table 4:** Regional Determinants in stunting prevalence evidence from BDHS 2011 and 2014, n = 13,378.

Covariates	Dhaka	Khulna	Rajshahi	Chittagong	Barisal	Rangpur	Sylhet
	Odds ratios (CIs)	Odds ratios (CIs)	Odds ratios (CIs)	Odds ratios (CIs)	Odds ratios (CIs)	Odds ratios (CIs)	Odds ratios (CIs)
**Time Dummy**							
DHS 2014	0.76*[0.60, 0.97]	0.83 [0.62, 1.10]	0.98 [0.77, 1.26]	1.18 [0.92, 1.51]	0.96 [0.71, 1.30]	0.89 [0.67, 1.17]	0.97 [0.72, 1.31]
**Age groups of child**							
12–23	3.98***[2.82, 5.63]	4.30***[2.63, 7.01]	1.60 [0.99, 2.61]	4.12***[2.50, 6.78]	2.93***[1.82, 0.74]	2.13***[1.37, 3.30]	3.64***[2.25. 5.89]
24–35	3.23***[2.30, 4.57]	2.12**[1.29, 3.51]	2.02**[1.30, 3.14]	5.05***[3.13, 8.17]	3.75***[2.39,5.90]	2.39***[1.54, 3.72]	5.03***[3.09, 8.20]
36–47	4.02***[2.71, 5.98]	3.00***[1.83, 4.90]	1.84*[1.13, 3.01]	5.97***[4.03, 8.83]	3.93***[2.53,6.09]	1.80***[1.23, 2.62]	5.44***[3.39, 8.71]
48–59	2.89***[1.97, 4.14]	2.02**[1.16, 3.50]	1.45 [0.87, 2.42]	4.00***[2.41, 6.63]	2.58***[1.66,4.02]	1.55*[1.01, 2.37]	3.44***[2.26, 5.21]
**Type of residence**							
Rural	0.74 [0.54, 1.02]	1.06 [0.79, 1.43]	0.81 [0.61, 1.06]	0.85 [0.64, 1.14]	0.91 [0.63, 1.30]	0.99 [0.71, 1.36]	1.01 [0.78, 1.32]
**Gender of child**							
Male	0.79**[0.66, 0.95]	1.19 [0.95, 1.49]	1.06 [0.82, 1.38]	1.14 [0.94, 1.37]	0.92 [0.70, 1.21]	1.33*[1.03, 1.70]	0.93 [0.74, 1.18]
**Religious affiliation of child**							
Non-Muslim	0.95 [0.57, 1.60]	0.65 [0.41, 1.04]	0.66 [0.36, 1.22]	0.99 [0.76, 1.28]	1.98**[1.24, 3.13]	0.93 [0.71, 1.21]	0.53**[0.34, 0.82]
**Birth order** ^a^	1.14**[1.05, 1.25]	1.12 [0.97, 1.28]	1.08 [0.96, 1.23]	1.05 [0.98, 1.11]	1.15** [1.03, 1.28]	1.01 [0.90, 1.12]	0.94 [0.86, 1.02]
**Mother’s age at birth**							
<20 years old	1.21 [0.93, 1.58]	1.28 [0.92, 1.78]	1.32*[1.00, 1.75]	1.01 [0.81, 1.25]	1.35 [0.98, 1.87]	1.18 (0.90, 1.55]	1.27 [0.94, 1.73]
**Mother’s BMI**							
< = 18.5 kg/m^2^	1.39 [0.97, 1.99]	1.45*[1.05, 1.99]	1.50***[1.21, 1.85]	1.38**[1.14, 1.68]	1.40* [1.04, 1.88]	0.97 [0.73, 1.30]	1.24 [0.95, 1.64]
**Mother’s height**							
< = 145 cm	2.32***[1.71, 3.14]	3.46***[2.35, 5.11]	3.26***[2.42, 4.39]	2.78***[2.15, 3.59]	2.13***[1.44, 3.17]	2.18***[1.58, 3.01]	2.09***[1.52, 2.87]
**Schooling years of Mother**							
Primary levels	1.05 [0.72, 1.52]	0.75 [0.47, 1.20]	1.02 [0.69, 1.51]	1.24 [0.92, 1.68]	0.96 [0.65, 1.40]	1.29 [0.94, 1.77]	0.91 [0.66, 1.27]
Secondary+	0.91 [0.61, 1.34]	0.88 [0.56, 1.38]	1.14 [0.78, 1.65]	0.97 [0.69, 1.37]	0.90 [0.58, 1.40]	1.14 [0.83, 1.56]	0.65* [0.45, 0.93]
**Schooling years of Father**							
Primary levels	0.92 [0.73, 1.15]	0.73 [0.52, 1.02]	0.87 [0.66, 1.14]	0.85 [0.66, 1.09]	0.78 [0.59, 1.03]	1.14 [0.83, 1.57]	0.91 [0.65, 1.29]
Secondary+	0.62***[0.46, 0.82]	0.76 [0.52, 1.11]	0.68*[0.48, 0.96]	0.52***[0.36, 0.76]	0.74 [0.47, 1.15]	0.80 [0.52, 1.24]	0.92 [0.63, 1.35]
**Household wealth quintals**							
2^nd^ quintile	0.91 [0.66, 1.24]	0.60**[0.40, 0.90]	1.09 [0.75, 1.59]	0.77 [0.54, 1.09]	0.61**[0.42, 0.87]	0.82 [0.60, 1.12]	0.48***[0.30, 0.75]
3^rd^ quintile	0.95 [0.69, 1.30]	0.60*[0.39, 0.91]	0.80[0.53, 1.20]	0.64** [0.48, 0.87]	0.56***[0.40, 0.78]	0.80 [0.57, 1.14]	0.47***[0.30, 0.71]
4^th^ quintile	0.72 [0.50, 1.03]	0.66 [0.42, 1.04]	0.56**[0.36, 0.88]	0.49***[0.36, 0.67]	0.46***[0.30, 0.70]	0.60**[0.41, 0.88]	0.34***[0.22, 0.52]
5^th^ quintile	0.40***[0.25, 0.63]	0.32***[0.18, 0.54]	0.42**[0.25, 0.73]	0.43***[0.26, 0.71]	0.25***[0.13, 0.48]	0.30***[0.18, 0.50]	0.17***[0.10, 0.29]
**Distance to nearest health clinics** ^a^ **(kilo.)**	1.01 [0.99, 1.04]	0.98 [0.94, 1.02]	0.99 [0.97, 1.02]	1.02***[1.01, 1.03]	0.98 [0.95, 1.01]	1.00 [0.97, 1.03]	0.99 [0.49, 1.72]

* p< = 0.05;

** p< = 0.01

*** p< = 0.001; 95% confidence intervals (CIs) are within bracket.

Reference category: Sylhet division, urban residence, 6–11 months old children, female child, religious affiliation of child is Muslim, survey year 2011, 0 year of schooling, mother’s BMI >18.5 kg/m^2^, mother’s height>145 cm, HH wealth quintile 1^st^

^a^ = birth order and distance to the nearest health clinics are entered as continuous variable

## Discussion

Regional indicators of child nutritional status are dissimilar in Bangladesh. The country has experienced remarkable change in child nutrition over the past few decades. Yet, few studies attempted to explore regional covariates of stunting and its change over time, using nationally representative data. The sustainable development goal (SDG) of ending all forms of malnutrition, including stunting by 2030, has been set internationally [6]. The Bangladesh Government’s Health, Population and Nutrition Sector Program aims to address the determinants of childhood stunting in Bangladesh, specifically taking into account the regional disparities [16]. This paper, with nationally representative BDHS 2011 and 2014 data, provided detailed regional level stunting prevalence over two periods and examined hypotheses of effects of socio-economic and other correlates of stunting among children under the age of five in Bangladesh as a whole and at regional levels by including time dummy and regional dummies as well. However, to ensure enough statistical power due to large sample, we considered results as our benchmark results (model 3 of Table 3 based on combined datasets). The paper applied statistical control for observed covariate effects and unobserved clustering effects. Thus, for better nutritional guidelines and region-specific interventions, the current research carried credence through highlighting some unique findings that may add valuable information in the literature of stunting in Bangladesh.

In 2014, little less than two fifths children were stunted in Bangladesh. Stunting among children under the age of five had declined substantially in all divisions of Bangladesh since 1996–97 especially in Dhaka, Rajshahi and Barisal divisions. However, since 2011, when all divisions reduced stunting at varying levels, no such decline was observed in Sylhet. Problem of growth failure and inadequate linear development of child with age and poor nutritional status of mother were foremost hindrances in stunting reduction across divisions. Bangladesh experienced more stunting of children with an increase in age, a typical feature of many developing countries, mainly due to micronutrient deficient food consumption. Also, the rich-poor gap of stunting remained high over time. In addition, urban vulnerable groups require focused attention to address stunting. Nevertheless, significant reductions of stunting in rural Bangladesh and in Dhaka division deserve appreciation. The lowest rate of stunting was observed in Khulna and Rajshahi divisions with some distinctive predictors elaborated in this section. Factors that played an imperative role in addressing regional difference in stunting were parental characteristics, like maternal health, father’s education and household wealth. In Bangladesh, father’s educational impact surpassed mother’s educational effect (see Table 3) on lowering the chances of stunting in certain divisions, while mother’s health (partly BMI kg/m^2^ and mainly height) played a vital role in improving child’s nutritional status. Being a patriarchal society, role of father in household wealth generation and his knowledge possibly are essential factors that can eliminate child undernutrition in Bangladesh. Regional intervention has the potential to be more efficient in addressing stunting because the determinants of stunting differ across administrative divisions in Bangladesh.

Literature strongly supports positive association of women’s health with decreasing levels of stunting in Bangladesh [17]. Malnutrition cycle is distinct in Bangladesh in almost all divisions, predominantly in Sylhet division. Stunting, as observed in our analysis, starts at the early childhood and it increases markedly with age, failing to catch up with age, typically giving rise to a short adolescent. Interventions that aim to break undernutrition cycle and to address mother’s short height are the need of the hour. Few children receive nutritionally adequate and safe complementary foods; in many countries, less than a fourth of infants 6–23 months meet the criteria of dietary diversity and feeding frequency that are appropriate for their age [18]. Saha *et al*. estimated that food security is positively associated with growth of young children [19]. Rahman et al, in 2016 [20], estimated from BDHS 2011 that children with low birth weight have significantly increased risk of becoming malnourished compared to their counterparts with relative risk (RR) of 1.23. Although we could not incorporate birth weight variable in our analysis due to data constraints in 2014 BDHS, it is confirmed from our study that short heighted mothers’ have a strong probability of having a stunted child in all divisions of Bangladesh.

Rich- poor gap of stunting though has reduced with time, needs attention. Our finding on rich-poor differences in the odds of stunting supports the evidence of higher rate of malnutrition among the children under the age of five from the poorest class and persistence of income inequality as one of the explanations for rich- poor divide in stunting [21–22].

It is appreciable that Bangladesh has been successful in addressing undernutrition problem of two sub population quite efficiently in a short span of 2011 to 2014. First, the odds of stunting in rural areas had reduced over time. Second, Dhaka division made commendable improvement in child nutrition and immensely contributed to the reduction of the overall levels of stunting in Bangladesh. This division had also effectively addressed the nutritional differentials of urban-rural areas, by wealth, birth order (5+), and mother’s health which were evident from descriptive statistics. Interestingly about 20% reduction in the prevalence of stunting was observed over the period, especially for the short heighted mothers in Dhaka.

The progress in the provision of basic amenities in Bangladesh is one of the fastest in South Asia and has been efficient in reaching the poorest communities [23] and tackling child undernutrition in the past. Nutrition has been mainstreamed and scaled up through the National Nutrition Services and linkages are made with local governments to ensure primary health care, including nutrition services [24]. Such initiatives perhaps explain improvement of child nutrition in the country as a whole, and specifically in Dhaka division, as evidenced in our findings. However, urban Bangladesh witnessed a growing incidence of child malnutrition and greater nutritional inequalities with worsening health indices due to poor environmental conditions [25–27]. Although Bangladesh has experienced improvement in providing basic amenities by addressing the practice of open defecation and through provision of safe drinking water supply [28–30], urban vulnerable groups require focused consideration.

Each division has its own distinctive factors that explain stunting, apart from the improvement in mother’s health and economic status. Two regions that require special attention to curb stunting are Sylhet and Barisal. National food security data has placed Sylhet as a relatively food secure region and yet this region has the poorest nutritional status [31]. Although Sylhet is a relatively rich district, poverty coexists with richness [25, 27], which is in line with our finding that maximum difference in stunting was estimated between the poorest-richest households in Sylhet where local geography is also not favourable for year-long agricultural production due to environmental shocks [31].

In our study, the odds in stunting for children in Barisal did not vary significantly from that of Sylhet. In these two regions, the odds of stunting increased with the attainment of higher education by the fathers. This finding contrasted the traditional determinants of childhood stunting in Bangladesh where father’s education played a stronger role to reduce the chances of stunting. It perhaps implies a deep rooted patriarchal society where educated fathers are playing restrictive role in child nutrition. Such condition can only be offset through mother’s education and nutritional knowledge dissemination to parents. In Sylhet, stunting was significantly less for those mothers with higher education, among richest quintile and in non-Muslim households. Poorest households were also more vulnerable in terms of their child growth in Chittagong and Rangpur divisions. However, unlike Sylhet, the reduced stunting levels in Chittagong and Rangpur can perhaps be explained by unobserved infrastructural development or by observed factors like reduced levels of poorest-richest differences in the stunting levels. Thus, apart from improving economic condition, other key interventions to address stunting are improving the health and education levels, especially in Sylhet.

The lowest rate of stunting was witnessed in the south-western-eastern region of Bangladesh, i.e. in Khulna and Rajshahi divisions. Khulna points to possibility of two explanations: Firstly, Khulna indicated no significant impact of mother’s education on stunting. Secondly, some experimental home gardening interventions implemented by BRAC in Khulna proved that women with nutrition education and gardening training can support households to produce and consume more vegetables which leads to improved intake of micronutrients [32]. Thus, Khulna substantiates that irrespective of educational standard, practical knowledge on nutrition and locally known food can also address stunting. Our results suggested (see Tables 1 and 2) that the households belonging to richest quintile in Khulna and Rajshahi had less stunted children in 2011 compared to other divisions which perhaps played a significant role in the reduced odds of stunting in these two divisions. In addition, there have been enough evidence of women’s autonomy and decision-making capacity in these regions [33–35] and thus they excel in child nutrition when compared to the other divisions of Bangladesh. It points out evasively the fact that knowledge on feeding practices and diverse food habits can tackle undernutrition. For example, our results for Rajshahi showed no significant differences in the odds of stunting with child’s age, which perhaps specifies better complimentary feeding practices in Rajshahi. In addition, diffusion of agrarian and cultural practices between the two Bengali-speaking regions, i.e. West Bengal and Bangladesh, cannot be ignored. We can corroborate this argument with the fact that the bordering districts of southern West Bengal (i.e. 24 Parganas), that are adjacent to Khulna, and Rajshahi hold similar demographic and nutritional rates [36]. There are similarities in the nature of the soil in Khulna, Rajshahi and adjacent districts of (24 Parganas) West Bengal. These areas are covered with loamy or clay loam soil of the Gangetic plain that has good agricultural productivity, which may explain the relatively better food supply compared to Sylhet, that has silty soil [37–38]. Hence favourable climatic and agricultural environment along with women’s empowerment and knowledge related to child nutrition serve as primary factors to reduce the stunting among children, as compared to northern division of the country.

There are some limitations of the study. Looking into the study sampling distribution across 7 regions over two periods (S1 and S2 Tables), it reveals that sampling distribution varies over regions. Thus, adjusted weighted factors may explain some differences in stunting reductions especially for Dhaka where covariates play a minimum role in explaining the reduced level of stunting. Also, due to matching problem in two data sets, we had lost six observations for the distance variable which however, did not change our conclusion. Although, we adjusted the models for clustering effects, owing to omitted variable problems, our results may suffer from unobserved heterogeneity. For example, environmental factors or specific interventions can play a role in explaining regional differences in stunting among under-fives. The limitation of our analysis is that, we could not disentangle unobserved heterogeneity from the observed covariates effects.

## Conclusion

Bangladesh has addressed decline in stunting levels in the past. Further decline in stunting is possible through region-specific multipronged interventions, targeting children at risk [39–42]. Dhaka has made laudable improvement in child nutrition and immensely contributed to the reduction of the overall levels of stunting in Bangladesh. However, unlike Dhaka, no other regional division of Bangladesh has been able to reduce stunting significantly in the recent past (2011–2014). To attain the targeted SDG goal of reducing stunting by 2030, series of actions are necessary [43–44]. Sylhet and Barisal require strong push to improve nutritional status of children belonging to Muslim and economically poor households to break the cycle of malnutrition. Besides socio-economic progress, substantial improvement of mother’s health and better involvement of men in formal and nutritional education may help tackling stunting in these two critical divines of Bangladesh. Reduction of stunting in Sylhet also requires attention across population groups, besides economic and health improvement.

Overall, to address stunting, Bangladesh needs to focus on household wealth generation, improvement of maternal health, interventions on vulnerable groups including the children from poorest socio-economic strata or children in the urban areas and strengthening the role of father to improve their knowledge in varying aspects like family planning, reduction of fertility, child nutrition and maternal care. Further, a targeted multi-sectoral programme is essential to reduce the prevalence of short heighted girls at an early age, to progress knowledge on complementary food for young children and to reinforce family planning program, aiming to increase the age at birth and to decrease higher order births, in order to achieve the SDGs by 2030.

## Supporting information

S1 Table Weighted percentage distribution of sample characteristics by different divisions of residence in Bangladesh, BDHS 2011. (DOC)

S2 Table Weighted percentage distribution of weighted sample characteristics by different divisions of residence in Bangladesh, BDHS 2014. (DOC)
