# Correction: Trends, prevalence and determinants of childhood chronic undernutrition in regional divisions of Bangladesh: Evidence from demographic health surveys, 2011 and 2014

**DOI:** 10.1371/journal.pone.0229677

**Published:** 2020-02-20

**Authors:** Unnati Rani Saha, Aparajita Chattopadhyay, Jan Hendrik Richardus

The second author’s name is spelled incorrectly. The correct name is: Aparajita Chattopadhyay. The correct citation is: Saha UR, Chattopadhyay A, Richardus JH (2019) Trends, prevalence and determinants of childhood chronic undernutrition in regional divisions of Bangladesh: Evidence from demographic health surveys, 2011 and 2014. PLoS ONE 14(8): e0220062. https://doi.org/10.1371/journal.pone.0220062
