# Supplementary material for: Trends, prevalence and determinants of childhood chronic undernutrition in regional divisions of Bangladesh: Evidence from demographic health surveys, 2011 and 2014
Source: PLoS One. 2019 Aug 9;14(8):e0220062. doi: 10.1371/journal.pone.0220062 (PMC6688800; doi:10.1371/journal.pone.0220062)
Supplement: S1 Table — (DOC) [file pone.0220062.s001.doc]

**S1 Table : Weighted percentage distribution of sample characteristics by different divisions of residence in Bangladesh, BDHS 2011**

| **Variables** | **Dhaka, n=1,160** | **Khulna, n=794** | **Rajshahi, n=836** | **Chittagong, n=1,372** | **Rangpur, n= 910** | **Barisal, n= 778** | **Sylhet, n=1,121** | **Total**  **N=6971** |
| --- | --- | --- | --- | --- | --- | --- | --- | --- |
| **Stunting (HAZ<-2 SD) age 6-59 months old children** | 45.58 | 35.48 | 35.37 | 44.36 | 43.95 | 46.37 | 52.32 | 44.48 |
| **Gender of child** |  |  |  |  |  |  |  |  |
| Male | 51.40 | 48.75 | 51.11 | 49.53 | 52.17 | 51.31 | 50.75 | 50.73 |
| **Age of child (months)** |  |  |  |  |  |  |  |  |
| 6-11 | 10.85 | 10.63 | 11.31 | 12.92 | 12.33 | 12.05 | 11.21 | 11.61 |
| 12-23 | 20.27 | 22.70 | 22.68 | 21.48 | 17.96 | 20.20 | 18.87 | 20.61 |
| 24-35 | 20.21 | 22.02 | 18.76 | 20.26 | 20.63 | 19.42 | 21.20 | 20.29 |
| 36-47 | 25.61 | 23.30 | 24.36 | 21.84 | 26.06 | 26.29 | 23.96 | 24.35 |
| 48-59 | 23.06 | 22.34 | 22.90 | 23.50 | 23.02 | 22.04 | 24.77 | 23.14 |
| **Birth order** |  |  |  |  |  |  |  |  |
| 1 *** | 34.96 | 42.62 | 33.98 | 31.94 | 35.56 | 37.50 | 25.71 | 34.35 |
| 2 *** | 28.07 | 33.26 | 34.27 | 27.50 | 31.67 | 28.68 | 24.02 | 29.31 |
| 3-4 *** | 27.19 | 19.99 | 24.57 | 28.41 | 26.87 | 24.35 | 32.97 | 26.73 |
| 5+ *** | 9.78 | 4.14 | 7.18 | 12.15 | 5.90 | 9.48 | 17.30 | 9.61 |
| **Religion** |  |  |  |  |  |  |  |  |
| Muslim**** | 95.98 | 88.94 | 93.82 | 91.96 | 81.42 | 92.56 | 84.29 | 91.46 |
| **Type of residence** |  |  |  |  |  |  |  |  |
| Rural *** | 66.82 | 79.75 | 84.24 | 78.44 | 88.46 | 85.33 | 88.01 | 77.86 |
| **Schooling years of Mother** |  |  |  |  |  |  |  |  |
| No education*** | 21.49 | 8.17 | 22.31 | 20.13 | 22.73 | 12.19 | 30.31 | 20.36 |
| Primary* | 33.11 | 28.61 | 30.86 | 26.63 | 29.21 | 35.86 | 35.54 | 30.86 |
| Secondary +*** | 45.40 | 63.21 | 46.83 | 53.24 | 48.06 | 51.95 | 34.14 | 48.77 |
| **Mother’s age at birth** |  |  |  |  |  |  |  |  |
| <20 years old *** | 31.11 | 34.97 | 32.22 | 29.66 | 39.41 | 32.76 | 20.97 | 31.50 |
| **Mother’s height** |  |  |  |  |  |  |  |  |
| <=145 cm | 12.83 | 8.39 | 12.95 | 13.00 | 14.95 | 12.12 | 12.67 | 12.65 |
| **Mother’s BMI** |  |  |  |  |  |  |  |  |
| <=18.5 kg/m^2 **^ | 29.93 | 23.89 | 31.04 | 28.84 | 34.13 | 32.16 | 40.76 | 30.69 |
| **Schoolings years of Father** |  |  |  |  |  |  |  |  |
| No education*** | 31.34 | 21.78 | 34.96 | 27.54 | 32.02 | 21.15 | 38.31 | 30.10 |
| Primary | 28.26 | 28.89 | 26.61 | 28.65 | 31.76 | 34.21 | 29.85 | 29.03 |
| Secondary + ** | 40.40 | 49.40 | 38.15 | 43.64 | 36.17 | 44.47 | 31.85 | 40.77 |
| **Household wealth quintals** |  |  |  |  |  |  |  |  |
| 1^st^ quintile | 20.77 | 21.77 | 23.60 | 22.37 | 20.58 | 22.22 | 23.27 | 21.83 |
| 2^nd^ quintile | 20.78 | 21.92 | 23.09 | 21.14 | 21.89 | 23.10 | 22.38 | 21.62 |
| 3^rd^ quintile | 20.73 | 20.62 | 20.44 | 20.65 | 22.52 | 21.40 | 21.26 | 20.94 |
| 4^th^ quintile | 19.58 | 19.53 | 19.11 | 19.43 | 19.87 | 19.22 | 18.52 | 19.41 |
| 5^th^ quintile | 18.15 | 16.16 | 13.76 | 16.41 | 15.23 | 14.06 | 14.58 | 16.20 |
| **Average distance to the nearest health clinics (km)** | 6.25 (4.48) | 5.92 (3.80) | 6.30 (4.00) | 6.98 (7.37) | 6.24 (3.67) | 6.35 (5.51) | 6.45 (5.32) | 6.41 (5.22) |
