# Supplementary material for: Trends, prevalence and determinants of childhood chronic undernutrition in regional divisions of Bangladesh: Evidence from demographic health surveys, 2011 and 2014
Source: PLoS One. 2019 Aug 9;14(8):e0220062. doi: 10.1371/journal.pone.0220062 (PMC6688800; doi:10.1371/journal.pone.0220062)
Supplement: S2 Table — (DOC) [file pone.0220062.s002.doc]

**S2 Table : Weighted percentage distribution of weighted sample characteristics by different divisions of residence in Bangladesh, BDHS 2014**

| **Variables** | **Dhaka n=1,120** | **Khulna n=711** | **Rajshahi n=812** | **Chittagong, n=1,215** | **Rangpur n= 791** | **Barisal n= 737** | **Sylhet n=1,021** | **Total**  **N=6407** |
| --- | --- | --- | --- | --- | --- | --- | --- | --- |
| **Stunting (HAZ<-2 SD) age 6-59 months old children** | 35.35 | 29.69 | 32.60 | 40.85 | 39.45 | 42.05 | 52.00 | 38.20 |
| **Gender of child** |  |  |  |  |  |  |  |  |
| Male | 51.95 | 51.36 | 50.45 | 50.80 | 51.59 | 52.43 | 51.21 | 51.42 |
| **Age of child (months)** |  |  |  |  |  |  |  |  |
| 6-11 | 13.05 | 12.20 | 13.03 | 12.56 | 10.69 | 11.79 | 12.32 | 12.50 |
| 12-23 * | 25.61 | 24.01 | 22.47 | 23.31 | 21.21 | 23.49 | 18.30 | 23.40 |
| 24-35 * | 19.81 | 23.83 | 19.75 | 23.50 | 22.59 | 22.37 | 22.69 | 21.60 |
| 36-47 | 21.41 | 20.68 | 21.93 | 21.05 | 21.20 | 19.17 | 22.17 | 21.26 |
| 48-59 * | 20.13 | 19.28 | 22.82 | 19.58 | 24.31 | 23.18 | 24.52 | 21.25 |
| **Birth order** |  |  |  |  |  |  |  |  |
| 1 * | 38.55 | 41.09 | 40.59 | 34.91 | 39.47 | 40.44 | 31.26 | 37.68 |
| 2 *** | 29.51 | 37.06 | 34.00 | 29.26 | 35.61 | 27.04 | 21.83 | 30.23 |
| 3-4 | 25.42 | 19.25 | 22.04 | 27.42 | 21.24 | 23.41 | 26.38 | 24.58 |
| 5+ *** | 6.52 | 2.59 | 3.37 | 8.41 | 3.68 | 9.11 | 20.52 | 7.51 |
| **Religion** |  |  |  |  |  |  |  |  |
| Muslim | 95.61 | 88.46 | 93.59 | 86.79 | 88.54 | 89.11 | 90.01 | 91.36 |
| **Type of residence** |  |  |  |  |  |  |  |  |
| Rural * | 67.38 | 75.27 | 81.47 | 72.48 | 86.67 | 77.67 | 85.41 | 74.82 |
| **Schooling years of Mother** |  |  |  |  |  |  |  |  |
| No education *** | 19.16 | 8.18 | 15.19 | 14.29 | 14.59 | 11.47 | 26.43 | 16.68 |
| Primary *** | 27.61 | 24.47 | 27.16 | 24.00 | 26.41 | 33.63 | 37.58 | 27.74 |
| Secondary + *** | 53.23 | 67.35 | 57.66 | 61.71 | 59.00 | 54.90 | 35.99 | 55.57 |
| **Mother’s age at birth** |  |  |  |  |  |  |  |  |
| <20 years old *** | 30.91 | 33.43 | 34.80 | 29.70 | 25.55 | 29.09 | 22.62 | 30.84 |
| **Mother’s height** |  |  |  |  |  |  |  |  |
| <=145 cm *** | 13.50 | 9.25 | 13.35 | 9.32 | 14.79 | 11.52 | 18.15 | 12.58 |
| **Mother’s BMI** |  |  |  |  |  |  |  |  |
| <=18.5 kg/m^2 **^ | 22.33 | 19.71 | 28.10 | 21.10 | 31.13 | 27.57 | 31.14 | 23.60 |
| **Schoolings years of Father** |  |  |  |  |  |  |  |  |
| No education *** | 29.08 | 19.17 | 29.23 | 19.92 | 24.39 | 17.49 | 41.11 | 26.44 |
| Primary * | 26.87 | 28.70 | 31.28 | 29.97 | 29.07 | 37.69 | 34.17 | 29.68 |
| Secondary + *** | 44.05 | 52.13 | 39.49 | 50.11 | 46.54 | 44.81 | 24.72 | 43.88 |
| **Household wealth quintals** |  |  |  |  |  |  |  |  |
| First | 24.46 | 22.17 | 23.57 | 22.87 | 18.79 | 22.32 | 24.32 | 23.15 |
| Second | 21.98 | 22.05 | 22.06 | 20.72 | 23.55 | 20.60 | 23.60 | 21.96 |
| Third | 20.23 | 20.35 | 21.48 | 20.72 | 18.73 | 20.10 | 19.19 | 20.22 |
| Fourth | 17.36 | 17.32 | 17.90 | 17.86 | 21.15 | 18.10 | 17.46 | 17.95 |
| Fifth | 15.96 | 18.11 | 14.99 | 17.83 | 17.78 | 18.88 | 15.44 | 16.72 |
| **Average distance to the nearest health clinics (km)** | 5.01 (3.84) | 5.57 (3.78) | 5.57 (4.42) | 5.37 (4.61) | 6.08 (3.51) | 5.53 (4.03) | 5.60 (4.07) | 5.41 (4.09) |
